# Fosfomycin Resistance: An Update on the Anthropogenic Impact Through Agriculture

**DOI:** 10.3390/pathogens15010029

**Published:** 2025-12-24

**Authors:** Weronika Goraj, Paweł Kowalczyk, Grzegorz Bełżecki, Adam Furtak, Anna Pytlak, Anna Szafranek-Nakonieczna

**Affiliations:** 1 Department of Microbiology and Translational Medicine, The John Paul II Catholic University of Lublin, Konstantynów 1I, 20-708 Lublin, Poland; 2 Department of Animal Nutrition, The Kielanowski Institute of Animal Physiology and Nutrition, Polish Academy of Sciences, Instytucka 3, 05-110 Jabłonna, Poland; p.kowalczyk@ifzz.pl (P.K.); g.belzecki@ifzz.pl (G.B.); 3 Department of Natural Environment Biogeochemistry, Institute of Agrophysics, Polish Academy of Sciences, Doświadczalna 4, 20-290 Lublin, Poland; a.furtak@ipan.lublin.pl (A.F.); a.pytlak@ipan.lublin.pl (A.P.)

**Keywords:** fosfomycin, antibiotic resistance, soil microbiota, resistome, animal husbandry, soil pollution, microplastics, pesticides, heavy metals, fertilisation

## Abstract

The extensive and often inappropriate use of antibiotics has led to the rapid emergence and spread of antibiotic resistance, reducing their effectiveness against pathogenic microorganisms. Fosfomycin has become an increasingly important therapeutic option in both human and veterinary medicine, particularly when other antibiotics fail. This review summarises current knowledge on the occurrence of fosfomycin resistance and evaluates the role of agricultural practices in its dissemination. Multiple microbial resistance mechanisms have been identified, including genes from the *fosA*, *fosB*, and *fosC* families, and new determinants continue to be reported. Agriculture contributes to the environmental spread of resistance through the use of antibiotics in food-producing animals, the exchange of resistant microorganisms between humans and animals, and the application of manure as fertiliser. Fosfomycin resistance genes have been detected in livestock such as pigs, chickens, pigeons, and cows, as well as in vegetables. Their presence in soil is influenced by fertilisation, nitrogen levels, microplastics, heavy metals, and pesticide application. Additionally, climate warming may facilitate the broader dissemination of fosfomycin resistance. Despite increasing evidence, current understanding remains limited. Further research is needed to elucidate the mechanisms driving the spread of fosfomycin resistance in agricultural environments and to develop effective monitoring strategies.

## 1. Introduction

As the human population grows, so does the threat posed by infectious diseases. This trend is driven not only by increased population density, which facilitates the transmission of pathogens, but also by the growing problem of antibiotic resistance. over the past decade, the number of deaths from untreatable infections has risen significantly [1,2]. New antibiotics offer a potential solution to this problem. Unfortunately, according to the latest World Health Organisation (WHO) report, the development of new antibacterial agents remains insufficient. In 2025, research was conducted on 90 new antibiotics—a decrease of seven compared to 2023. Furthermore, of the substances studied, only five are effective against critical bacteria and only 15 are considered innovative, meaning they represent a new chemical class, target a novel bacterial mechanism, operate via a new mode of action, or exhibit no known cross-resistance with existing drugs [3]. This lack of innovation reduces the likelihood of a achieving real breakthrough in the treatment of difficult infections and the fight against multi-antibiotic-resistant pathogens.

Currently, one of the few available treatments for severe drug-resistant bacterial infections is fosfomycin [(2R,3S-3-methyloxiran-2-yl) phosphonic acid]. This naturally derived antibiotic has a broad spectrum of activity and, importantly, is effective against infections caused by critical pathogens, including multidrug-resistant (MDR) strains of *Escherichia coli*, *Klebsiella pneumoniae*, and *Enterobacter* species, which are major causes of hospital-acquired infections It also shows good activity against methicillin-resistant *Staphylococcus aureus* (MRSA) and vancomycin-resistant *Enterococcus faecium* (VRE) [4]. For this reason, fosfomycin is included in the WHO list of medically important antimicrobials for human medicine and classified as a “Highest Priority Critically Important Antimicrobial” for human medicine [5].

Fosfomycin is used in both human and veterinary medicine [6]. The World Organisation for Animal Health (WOAH) aligns its guidance with that of the WHO to promote a “One Health” approach to antimicrobial resistance. The WOAH List of Antimicrobial Agents of Veterinary Importance classifies phosphonic acid derivatives (e.g., fosfomycin) as Highest Priority Critically Important Antimicrobials (HPCIA) [7].

In human medicine, fosfomycin remains a clinically important therapeutic option. Oral formulations are widely used as a first-line treatment for uncomplicated urinary tract infections, while intravenous fosfomycin is increasingly employed as part of combination therapy for severe infections caused by multidrug-resistant Enterobacterales and *Staphylococcus aureus* [8]. Its designation as a Highest Priority Critically Important Antimicrobial (HPCIA) reflects the recommendations of the FAO/WOAH/WHO Tripartite (now Quadripartite: FAO/WOAH/WHO and UNEP) collaboration, which provides harmonised global guidance on antimicrobial stewardship and the protection of agents essential for human health [9,10,11]. Within this framework, fosfomycin is considered an antimicrobial whose use in animals should be restricted or avoided whenever possible, in order to preserve its efficacy in human medicine [12,13]. These international guidelines underscore the need to assess agricultural and environmental drivers of resistance through a One Health perspective.

Unlike previous reviews, our work integrates molecular mechanisms of fosfomycin resistance with environmental co-selective pressures arising from agricultural practices, including the effects of pesticides, microplastics, heavy metals, and global warming. This synthesis offers a novel One Health perspective on the emergence and dissemination of fosfomycin resistance.

The critical importance of fosfomycin calls for targeted measures to prevent the development of resistance to it. One of the primary concerns in this area is the intensification of agriculture and animal husbandry. This article presents the current state of knowledge on the mechanisms of fosfomycin resistance and the role of agriculture in its dissemination. We also identify existing knowledge gaps and propose research directions that could contribute to the acquisition of new insights useful for efforts to limit the spread of fosfomycin resistance.

## 2. Fosfomycin—History and Application

The discovery of fosfomycin (originally named phosphonomycin) was announced in 1969 and resulted from extensive research aimed at identifying substances that inhibit the formation and growth of bacterial cell walls. The microorganisms that it was first isolated from were strains of *Streptomyces* (*S. fradiae* (ATCC 21096), *S. viridochromogenes* (ATCC 21240), and *S. wedmorensis* (ATCC 21239) [14]. In subsequent years, it was also found to be produced by *Pseudomonas viridiflava* PK-5 and *Pseudomonas syringae* PB-5123 [15,16].

The chemical synthesis of fosfomycin, enabling its commercial production, was developed as early as 1969 [17]. However, the antibiotic did not gain widespread use. Its limited application after discovery was due, among other things, to the fact that early preparations containing fosfomycin sodium were approved only for parenteral administration in hospital settings and were not authorised for use in many countries. In subsequent years, oral formulations containing fosfomycin calcium were developed, but the poor absorption of the active substance (20–30%) remained a limitation, reducing its effectiveness in the treating infections outside the digestive tract. Broader use, particularly for the treatment of urinary tract infections, began in the 1990s and with the introduction of oral preparations containing fosfomycin trometamol, which offered higher absorption (approximately 40%). By that time, however, there were already many other antibiotics on the market, and fosfomycin remained primarily used in the treatment of urinary tract infections [18,19,20].

The first decade of the 21st century, brought renewed interest in the use of fosfomycin for clinical treatment. This was due to its broad spectrum of activity, limited resistance among critical pathogens resulting from prior ‘under-exploitation’, and its effectiveness against infections caused by multidrug-resistant bacteria [18,20,21,22].

## 3. Chemical Structure of Fosfomycin and Its Implications for Environmental Persistence

**Figure 1:**
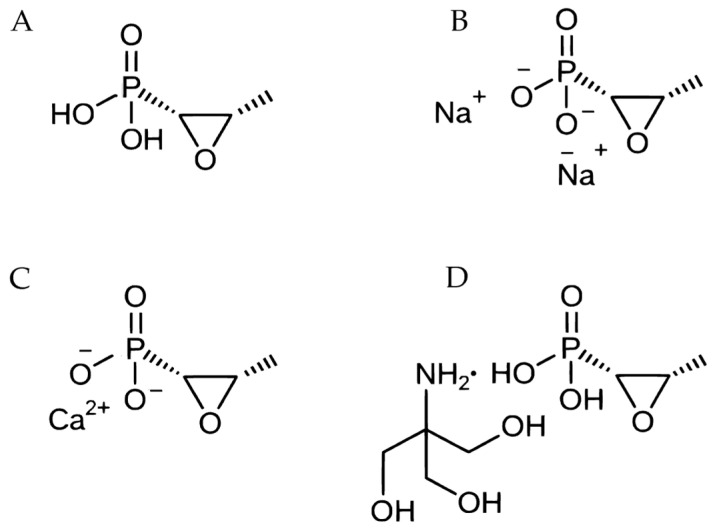
Chemical structure of fosfomycin (**A**), fosfomycin, disodium (**B**), fosfomycin calcium (**C**), and fosfomycin trometamol (**D**). The dot represents ionic interactions between fosfomycin and trometamol [20].

Fosfomycin is the only antibiotic classified as an organophosphonate (OPs), a group of compounds characterised by the presence of a carbon–phosphorus (C-P) bond [20,23]. This bond is high stable, making fosfomycin, like other organophosphonates, resistant to abiotic degradation caused by, for example, elevated temperatures, the presence of strong acids or bases, and photolysis [23]. The C-P binding also means that, despite its structural similarity to phosphate esters and anhydrides, and similarly to other OPs, fosfomycin is not susceptible to enzymatic degradation by phosphatases and phosphodiesterases, which are commonly found in the environment [20]. Instead, degradation/inactivation of fosfomycin under natural conditions requires the action of specific enzymes which target its epoxy ring (Figure 1), such as FosA, FosB, and FosX.

The biodegradation time of fosfomycin in soil remains unknown. In general, only a limited number of microorganisms are capable of cleaving the C–P bond and metabolising OPs, resulting in the overall low biodegradation rates of these compounds under both aerobic and anaerobic conditions [23].

Studies on wastewater microbial communities indicate that fosfomycin undergoes slow degradation/deactivation in the environment [24]. In addition to the presence of the C–P bond, this slow biodegradation of fosfomycin may also be attributed to the compound’s strong polarity, a characteristic feature of OPs. This strong polarity leads to significant adsorption onto sludge (and potentially also soil) particles, thereby reducing the compound’s bioavailability [23]. It is estimated that between 20% and 80% of total OPs in wastewater treatment plant (WWTP) influent are present in an adsorbed form [25]. Rather than engaging in fosfomycin degradation, exposed microbial communities often exhibit accelerated ecological and functional remodelling, which alters selective regime and drives the preferential expansion of resistant lineages [24].

Both the environmental persistence of fosfomycin and its substantial impact on microbial community composition are concerning, as they collectively exacerbate the proliferation of resistance within ecosystems contaminated by this antibiotic.

## 4. Mechanisms of Resistance to Fosfomycin

**Figure 2:**
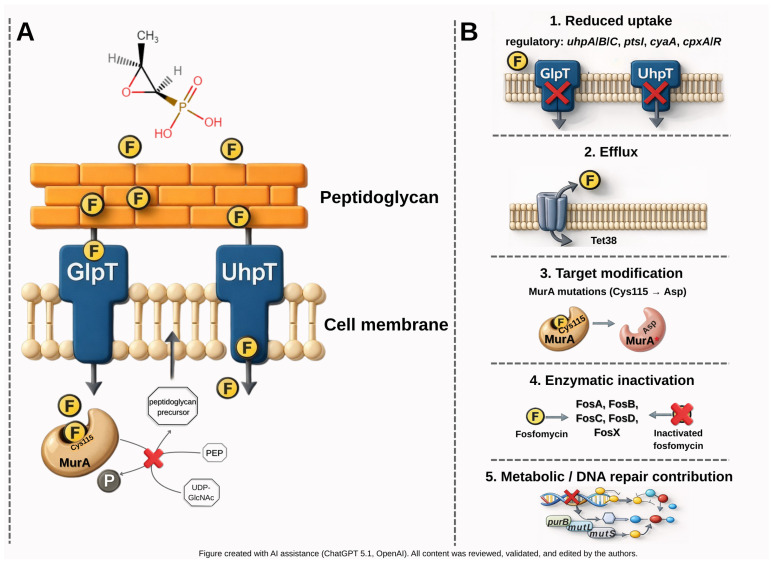
Uptake and mechanism of action of fosfomycin (fosfomycin—yellow circles) (**A**) and major mechanisms contributing to fosfomycin resistance (**B**), MurA* denotes a mutated form of MurA with reduced affinity for fosfomycin.

Fosfomycin was found to inhibit peptidoglycan biosynthesis in both Gram-positive and Gram-negative bacteria. Its mechanism of action involves blocking cell wall precursor synthesis catalysed by the MurA enzyme (UDP-N-acetylglucosamine enolpyruvyl transferase). In the absence of the antibiotic, UDP-N-acetylglucosamine (UDP-GlcNAc) reacts with phosphoenolpyruvate (PEP) to form UDP-GlcNAc-enoylpyruvate and inorganic phosphate. Fosfomycin acts as a PEP analogue that binds covalently (irreversibly) to the active site of MurA, thereby preventing the formation of UDP-N-acetylmuramic acid, ultimately leading to bacterial cell death (Figure 2A) [20].

Resistance to fosfomycin can arise through several mechanisms (Figure 2B). These include enzymatic modification of fosfomycin, changes in the structure of the MurA active site, and alterations in the fosfomycin transport system into the cell. Furthermore, recent studies highlight the contribution of cellular processes (e.g., central metabolism, nucleotide synthesis, and DNA repair) to the development of fosfomycin resistance.

*S. wedmorensis*, a natural producer of fosfomycin, inactivates the antibiotic through the production of FomA and FomB kinases (encoded by the *fomA* and *fomB* genes) [26]. These enzymes catalyse the phosphorylation of fosfomycin and fosfomycin monophosphate, respectively, enabling self-resistance in this fosfomycin-producing organism.

Fosfomycin-modifying enzymes that confer resistance and are found in other microorganisms include, for example: FosA (a glutathione S-transferase common in Gram-negative bacteria), FosB (a bacillithiol or L-cysteine transferase typical of Gram-positive bacteria), FosC and FosD (Mn^2+^-dependent thiol transferases), and FosX (an epoxide hydrolase).

There are many variants of *fosA* genes (*fosA1–fosA13*) that encode glutathione S-transferase. This enzyme catalyses the addition of a glutathione moiety to the epoxide ring of fosfomycin, resulting in its inactivation. FosA was originally discovered on a plasmid in *Serratia marcescens* [27]. According to current knowledge, *fosA* genes occur both on the chromosome and on plasmids, and are widely distributed among Gram-negative bacteria, especially in *Klebsiella* and *Escherichia coli* [28,29,30,31]. Some fosfomycin-resistant isolates, for example, *E. coli*, harbour transferable *fosA* genes and are also producers of extended-spectrum beta-lactamases (ESBLs). In such cases, treatment options such as fosfomycin and amoxicillin-clavulanic acid are ineffective [30,32,33,34]. It has also been reported that *K. pneumoniae* can harbour the colistin resistance gene *mcr-10* in addition to *fosA5* [35]. Moreover, the first *fosA3*-carrying cointegrate plasmid, pHN7A8–IncX3–type, identified in *E. coli* isolates and also capable of carrying carbapenem resistance genes (e.g., *blaNDM*), was recently described [31].

The next group, *fosB* genes, encodes Mg^2+^- or Mn^2+^-dependent thioltransferases that inactivate fosfomycin by adding bacillithiol to the antibiotic. These genes are typically found in Gram-positive bacteria, including *Staphylococcus*, *Enterococcus*, and *Bacillus* [36,37,38].

The *fosC* and *fosD* genes, similar to *fosB*, encode Mn^2+^-dependent thioltransferases. They are characteristic of certain species, such as Aeromonas (*fosC*) and *Staphylococcus* (*fosD*), respectively [39,40].

FosX enzymes, in turn, found in *Listeria monocytogenes*, *Clostridium botulinum*, and *Brucella melitensis*, are associated with *fosX* genes that encode Mn^2+^-dependent epoxidases. These enzymes use water to cleave the epoxide ring of fosfomycin, leading to its inactivation [38].

In addition to these well-described mechanisms, there are also reports of other chromosomal genes associated with high-level fosfomycin resistance—*fosK*, in *E. coli* [41] and *fosM* (similar to *fosB*), identified in human gut bacteria [42]. However, to date, these genes have only been reported in single studies.

Another mechanism of fosfomycin resistance involves mutations in the *murA* gene. Amino acid substitutions—such as the replacement of cysteine with aspartate—alter the conformation of the MurA active site, rendering it inaccessible to fosfomycin while preserving its function in cell wall synthesis. Several studies have documented that fosfomycin-resistant *Enterococcus* isolates harbouring *murA* mutations frequently also carry *fosB* genes.

Resistance may also be caused by alteration of the expression of the *glpT* and *uhpT* genes, which encode fosfomycin transporters: GlpT(glycerol-3-phosphate transporter) and UhpT (hexose-6-phosphate transporter) Resistance can also arise from their regulatory genes (*uhpA, uhpB, uhpC, ptsI, cyaA, cpxA*, *cpxR*). The expression of these transporter systems depends on their respective substrates—glycerol-3-phosphate and hexose-6-phosphate—and is positively regulated by *cAMP*. Mutations in any of the regulatory genes in this system reduce the synthesis of fosfomycin transporters, inducing resistance by preventing antibiotic transport into the cytoplasm.

Resistance may also arise from the active transport of fosfomycin out of the cell to prevent MurA damage. It has been shown that overexpression of the Tet38 efflux pump contributes to fosfomycin resistance in *S. aureus*.

Furthermore, recent genome-wide studies aimed at identifying chromosomal genes whose mutations enhance bacterial growth and survival in the presence of fosfomycin have revealed several new genes that modulate fosfomycin sensitivity. These genes are involved in pyruvate metabolism, the pentose phosphate pathway, nucleotide biosynthesis, DNA repair, protein translation, cellular iron homeostasis, and biotin biosynthesis. Notably, deletion of DNA repair genes (*mutL* and *mutS*) and purine biosynthesis genes (i.e., *purB* and its upstream gene *hflD*) led to the most significant increase in fosfomycin resistance. These results indicate that fosfomycin resistance-related processes are also linked to central cellular metabolism.

A summary of known fosfomycin resistance genes is provided in Table 1.

## 5. Agriculture’s Contribution to the Spread of Fosfomycin Resistance

### 5.1. Fosfomycinuse in Veterinary Medicine

The development of resistance may also be facilitated by the use of fosfomycin in livestock. Although WOAH advises against the veterinary use of this antibiotic, it continues to be employed in some regions. In the European Union, legislation governing fosfomycin is highly restrictive. Under Regulation (EU) 2019/6, its use in animals is generally not authorised, particularly in food-producing species [12].

However, regulatory frameworks differ considerably across countries. In China and India, fosfomycin is not included on the lists of substances prohibited for veterinary use. Nevertheless, no veterinary medicinal products containing fosfomycin are registered, nor have maximum residue limits been established for animal-derived food products. Consequently, the use of fosfomycin for the treatment or prevention of infections in animals is, in practice, illegal in these countries.

In Japan, intravenous fosfomycin has been approved for the treatment of bacterial diseases in cattle and horses, as well as for the management of infections in fish (primarily koi carp, trout, and salmon). The prophylactic use of antibiotics in healthy herds or aquaculture stock is permitted only under very limited, justified circumstances and strictly under veterinary supervision [63].

In Vietnam, fosfomycin is used mainly for the treatment of bacterial infections in poultry. Preventive use in feed was permitted until 31 December 2020, in accordance with [64].

In Argentina, fosfomycin was used primarily in poultry and swine until 2024. However, recent legislation has tightened regulations on antibiotic use, including fosfomycin. Under Regulation 445/2024 [65], products containing fosfomycin derivatives may only be used when alternative treatments have failed, and exclusively in animals producing products intended for export. Prophylactic use is not permitted, and the distribution of fosfomycin-containing products is now strictly regulated through an electronic prescription system [66].

In Brazil, fosfomycin is also approved for use in swine and poultry. Although its prophylactic application is not explicitly prohibited, the regulation governing medicated feed [67] (and subsequent amendments) imposes strict criteria on veterinary drugs incorporated into feed and restricts the use of medicated feed. Antibiotics—including fosfomycin—must be administered based on a clinical diagnosis and, whenever possible, supported by laboratory testing.

Despite substantial variation in national regulatory frameworks, a general trend toward the tightening of antibiotic-use policies has been observed in recent years, with gradual alignment toward WHO and WOAH recommendations. In countries where fosfomycin remains authorised for veterinary use, withdrawal periods for animal products such as meat and eggs range from 1 to 10 days, reducing the risk of antibiotic transmission through the food chain and limiting the development of antimicrobial resistance within the human gut microbiota.

Examples of fosfomycin resistance genes detected in agriculture-related samples are presented in Table 2.

### 5.2. Impact of Agricultural Practices on the Development of Resistance to Fosfomycin

#### 5.2.1. Fertilisation

One of the factors contributing to the increased prevalence of fosfomycin resistance genes is the use of organic fertilisers. Animal excreta serve as an important reservoir of these resistance genes [83]. When animal waste is applied to agricultural land as fertiliser, it can promote the dissemination of fosfomycin residues and antibiotic-resistant bacteria into soil ecosystems. As noted previously, fosfomycin exhibits limited gastrointestinal absorption and is only minimally metabolised. Consequently, treated animals excrete substantial quantities of the administered dose, and manure may contain both residual antibiotic and gut bacteria selected for fosfomycin resistance [84].

Notably, industrial farming practices promote the emergence of antibiotic resistance, even in animals that have not been subjected to antimicrobial treatment (but exposed, for example, to Zn treatment). This effect has already been described. For instance, research performed on swine manure from Bavarian farms demonstrated that despite the absence of fosfomycin treatment, the prevalence of *E. faecium* resistant to fosfomycin in pig slurry (83.1%) was more than fourfold higher than in municipal sewage sludge (24.8%) [83]. Similarly, analysis of cattle manure has shown a high abundance of fosfomycin resistance genes, even when animals were treated therapeutically with other antibiotics such as pirlimycin or cephapirin [85]. This is because, even in the absence of direct antibiotic exposure, agricultural practices can independently drive the emergence and dissemination of antibiotic resistance through physiological stress responses, de novo evolution, species sorting that favours resistant taxa, and elevated rates of horizontal transfer of antimicrobial resistance (AMR) genes [86].

Fosfomycin resistance is also frequently detected in bacterial isolates from commercial poultry farms across various regions of the world, including China, where the use of this antibiotic is formally prohibited [43,45,87]. Therefore, manure originating from intensive livestock production poses a risk of transmitting antibiotic residues (when antibiotics have been used), as well as resistant microorganisms, into the environment. Composting can reduce the concentration of drug residues [88]; however, studies by Chen et al. [85] demonstrated that the amendment of soils with cow manure—even when composted—led to an increase in the relative abundance of ARGs conferring fosfomycin resistance. However, the effect of organic fertilisation is soil- and microbiota-dependent, as other studies using dairy or swine manure reported no increase in fosfomycin resistance [89].

Mineral fertilisation has also been identified as a contributing factor. A link between the antibiotic resistome and microorganisms involved in the nitrogen cycle has been proposed [90], and increased frequencies of multiple AMS have been observed following the application of chemical fertilisers [90,91] as well as nitrogen-rich organic amendments [91]. AMR abundances were significantly correlated with nitrate/nitrite reduction genes, and Caulobacteraceae were identified as major hosts of both AMR and nitrogen-cycling genes in the studied soils [91]. These findings are consistent with results showing that elevated nitrate inputs into lake waters and sediments increased antimicrobial resistance associated with nitrate-reducing microbiota [92]. Given the close relationship between soil nitrogen and the distribution of AMR, soil nitrogen management may offer a means of mitigating risks associated with the soil antibiotic resistome.

At present, however, there is no information specifically addressing the impact of mineral fertilisation on fosfomycin resistance.

#### 5.2.2. Plastics

Plastics also represent a major environmental concern contributing to soil contamination and the spread of antimicrobial resistance. It has been reported that more than half of all residual microplastics in the environment are retained in soils [93]. A substantial proportion of these particles is introduced directly through agricultural activities, including the use of plastic mulches, seedling trays, and other plastic materials [94]. Sewage sludge applied as fertiliser constitutes another significant source of microplastics in soils [95].

The distinct environmental conditions created by plastics in soil have led to the introduction of the concept of the “plastisphere” [96]. The surfaces of plastics—including microplastics (typically <5 mm)—support the formation of specialised biofilms composed of microorganisms and extracellular polymeric substances. These structured microbial communities promote the persistence of antimicrobial-resistant bacteria and enhance the frequency of ARG exchange among bacteria [97,98], including through conjugative gene transfer. Plastics can also sorb extracellular plasmids released by bacteria, increasing plasmid stability and thereby raising the likelihood of horizontal transfer of ARGs to other microorganisms [99,100].

Research on the impact of smaller particles—nanoplastics (<100 nm) remains limited. However, current evidence suggests that due to their small size, NPLs may penetrate cells, induce the generation of reactive oxygen species, and increase cell membrane permeability, thereby facilitating the transfer of mobile genetic elements carrying antibiotic resistance genes (including fosfomycin resistance) within microbial communities [101].

Plastic additives such as bisphenol analogues (including bisphenol A) and the plasticiser dibutyl phthalate, used in polylactic acid, can further enhance the frequency of ARG transfer by inducing cellular stress [102,103].

Moreover, plastic surfaces readily adsorb environmental contaminants such as metals and pesticides [104], stressors whose effects on antimicrobial resistance has also been confirmed.

#### 5.2.3. Pesticides

The response of microbial antibiotic resistance to pesticides has recently been identified as an emerging threat. Pesticide-induced stress has been shown to enhance the acquisition of antibiotic resistance in bacteria through multiple mechanisms, including activation of efflux pumps, inhibition of outer membrane porins (leading to reduced antibiotic permeability), and the induction of gene mutations. Enhancement of horizontal gene transfer is considered the principal pathway through which pesticides influence the dissemination of antibiotic resistance genes (ARGs) in bacterial communities. Pesticides have also been shown to promote the conjugative transfer of ARGs by increasing cell membrane permeability and enhancing the abundance of mobile genetic elements involved in ARG propagation [105].

Importantly, glyphosate—one of the most widely used pesticides globally—appears to exert a particularly strong effect compared to other herbicides such as dicamba and glufosinate in increasing the abundance of fosfomycin resistance genes. In soil microcosms, a 60-day exposure to glyphosate resulted in a 14-fold increase in the abundance of fosfomycin resistance genes [106]. Field studies confirmed that soils from glyphosate-treated agricultural plots exhibited elevated fosfomycin resistance, whereas fallow soils showed no detectable resistance. A meta-analysis of pesticide-exposed soil metagenomes further demonstrated that the relative abundance of fosfomycin resistance genes was higher in soils amended with non-antibiotic pesticides (glyphosate, dicamba, glufosinate, prothioconazole, and chlorothalonil) than in soils exposed to antibiotic classes such as aminoglycosides, tetracyclines, and quinolones [107].

Glyphosate consistently increases the prevalence of fosfomycin resistance genes. Although glyphosate and fosfomycin are both organophosphonates, currently known fosfomycin resistance enzymes do not degrade fosfomycin at the structural position analogous to the glyphosate cleavage site (see Table 1). Thus, the underlying mechanism behind glyphosate-associated enrichment of fosfomycin resistance remains unknown and warrants further investigation. Given the widespread use of glyphosate and glufosinate (also an organophosphonate) [108,109,110] their potential role in enhancing intrinsic fosfomycin resistance deserves attention and should be incorporated into One Health monitoring frameworks and future research priorities.

#### 5.2.4. Heavy Metals

Agriculture is a major source of heavy metal contamination. Zinc and copper are routinely added to livestock feed to control infections and promote growth [111], and concentrations of Cu, Zn, and antibiotics reach particularly high values in piglet faeces [112]. In addition, heavy metals are common components of fertilisers, pesticides, and fungicides [86], and soils also accumulate heavy metals from atmospheric deposition, including dust and particulate matter [113].

Heavy metal pollution is particularly concerning because, unlike organic contaminants such as antibiotics or pesticides, metals do not degrade in the environment and can exert long-lasting and widespread selective pressure on microbial communities.

Genes encoding resistance to heavy metals are frequently genetically linked to antibiotic resistance genes, and plasmids, transposons, and integrons play key roles in the assembly and horizontal transmission of these combined resistance elements. Co-selection of metal and antibiotic resistance determinants has been repeatedly demonstrated, although the underlying mechanisms remain an active area of research [114,115].

With respect to fosfomycin, only a few studies have examined how heavy metal contamination influences the abundance of resistance genes. Available data indicate that soils contaminated with metals and radionuclides exhibit a 4–6-fold higher abundance of fosfomycin resistance genes compared with uncontaminated samples [116]. Similarly, in Zn- and Cd-contaminated soil, the addition of chitosan and *Trichoderma harzianum*—which increase metal bioavailability—resulted in an 87% increase in the abundance of fosfomycin resistance genes [117]. Metal- and fosfomycin-resistant bacteria have also been isolated from the green tissues of the heavy metal hyperaccumulator *Armeria maritima* [118].

However, an analysis of antibiotic resistance induction within manure microbiota showed that although heavy metals facilitated the proliferation of many ARGs through co-selection, this effect did not extend to fosfomycin [119]. This suggests that the induction of antibiotic resistance by heavy metals may depend on the specific composition of microbial communities and the minimum inhibitory concentrations (MICs) relevant to each compound, although this relationship requires further clarification.

#### 5.2.5. Global Warming

Global warming is currently one of the most pressing global challenges. Forecasts indicate that in the coming decades, atmospheric concentrations of greenhouse gases will continue to increase, leading to a rise in temperatures. Agriculture, primarily intensive livestock farming, and the associated emissions of CH_4_ and CO_2_, is one of the main contributors of this phenomenon and, at the same time, one of the areas of human activity that is most vulnerable to the consequences of climate change caused by global warming [120].

Noteworthy, rising global temperatures have been shown to increase the abundance and diversity of antibiotic resistance genes in soil, mainly by altering microbial communities and increasing the mobility and expression of resistance genes [86,121]. However, with regard to fosfomycin resistance, the evidence is not clear. Large-scale soil and metagenomic studies consistently indicate that warming enriches ARGs for several classes of antibiotics, but the effect on fosfomycin resistance genes is either insignificant or significantly weaker compared to other antibiotics, such as beta-lactams, tetracyclines and macrolides [122].

Additionally, studies conducted on polar and temperate soils confirm the presence of fosfomycin resistance genes in environmental resistomes, but show no clear increase in their prevalence due to warming. The overall trend indicates that although global warming may increase the overall risk of soil antibiotic resistance, its direct impact on fosfomycin resistance is less clear and may depend on local ecological and microbiological factors [122,123]. Certainly, the physicochemical properties of soil should be included in these considerations as a factor that is subject to change with climate change [124] and has a significant impact on the development of antibiotic resistance [125].

## 6. Methodological Advancements in the Study of Fosfomycin Resistance

The identification and characterisation of fosfomycin resistance genes have undergone substantial methodological evolution during the past few decades. Research approaches have shifted from cultivation-dependent and phenotypic assays to culture-independent, high-throughput molecular and omics-based strategies. This transformation has enabled the exploration of resistance determinants, not only in pathogenic bacteria, but also within complex environmental matrices such as soil, manure, and wastewater. Evaluating these methodological advancements is essential for assessing the anthropogenic dissemination of fosfomycin resistance within agricultural systems.

### 6.1. Classical and PCR-Based Methods

Early studies on fosfomycin resistance primarily relied on classical microbiological techniques, including selective cultivation and phenotypic testing [126], which could only capture a small fraction of the total microbial diversity. While these approaches provided valuable baseline information, they were limited by their inability to detect non-culturable organisms [127].

The introduction of polymerase chain reaction (PCR) and quantitative real-time PCR (qPCR) represented a pivotal advancement. These techniques enabled targeted detection of known fosfomycin resistance genes (*fosA*, *fosB*, *fosC*, *fosX*) directly from environmental DNA [68,128,129]. More recently, high-throughput qPCR (HT-qPCR) platforms have allowed the simultaneous quantification of hundreds of antimicrobial resistance genes (ARGs) in a single run, offering high-resolution insights into the resistome composition of soil and wastewater samples [129,130,131]. However, HT-qPCR still cannot assign genes to specific taxa or reveal gene expression dynamics [131,132]. Moreover, while PCR-based assays are rapid and cost-effective, they are inherently limited to genes with known sequences.

Furthermore, it is also worth noting that the importance of fosfomycin resistance has still not been given sufficient attention. An example of this is the widely used, standardised gene panel proposed by Berendonk [133]. The idea behind the suggested method was to enable global comparability of clinical and veterinary samples in order to track the spread of environmental antibiotic resistance. It includes a number of primers targeting resistance to critical antibiotics and markers of horizontal gene transfer, but contains no reference to fosfomycin.

It is also worth noting that laboratories in low-resource regions often face challenges in implementing even basic molecular diagnostics, underscoring the need for affordable and scalable diagnostic platforms.

### 6.2. Meta-Omics Approaches

The advent of high-throughput sequencing has revolutionised the study of antibiotic resistance in the environment. Shotgun metagenomics enables comprehensive profiling of microbial communities, including the detection of both known and novel resistance genes, and their association with mobile genetic elements. Recent metagenomic studies have revealed previously unrecognised *fosX*-like genes and chromosomal fosC3 variants in diverse environmental niches [39,52].

Complementary metatranscriptomic analyses, which assess total environmental RNA, provide insight into the active transcription of resistance genes under specific environmental conditions. This approach helps distinguish between dormant and active resistomes, highlighting genes that are transcriptionally expressed in situ [134,135,136].

Metaproteomics, which focuses on the full complement of proteins in environmental samples, adds another layer of information by identifying resistance mechanisms expressed at the protein level. Although technically demanding, it bridges the gap between genomic potential and actual phenotypic expression [134].

Although meta-omics approaches have transformed the study of antimicrobial resistance in environmental microbiomes, several methodological constraints remain. High-throughput sequencing (HTS) may fail to detect low-abundance resistance genes due to limited sequencing depth and background complexity, while quantitative accuracy is affected by variations in DNA extraction and library preparation. Moreover, reliance on incomplete or outdated reference databases can lead to the omission or misclassification of genes, particularly novel or divergent ones. Meta-omics analyses also often lack contextual information regarding the genetic location and host association of ARGs, making it difficult to assess their mobility and ecological risk. Finally, these approaches remain technically demanding and costly, which limits their routine use, especially in low-resource settings [137,138].

Addressing these limitations requires methodological standardisation, enhanced and regularly updated reference databases, and the integration of complementary techniques such as functional metagenomics and single-cell sequencing to validate and contextualise meta-omics findings.

### 6.3. Functional Metagenomics

Functional metagenomics remains one of the most powerful tools for discovering novel antibiotic resistance determinants. In this approach, fragments of environmental DNA are cloned into expression vectors and screened for phenotypic traits, such as the ability to grow in the presence of fosfomycin. This strategy enables the identification of entirely new resistance genes, even those without homology to known sequences—a crucial advantage over purely sequence-based methods [139,140]. Recent functional metagenomic studies have successfully identified four new *fosX*-family genes from environmental samples, illustrating the approach’s capacity to uncover previously hidden mechanisms of resistance [141].

Despite its strengths, functional metagenomics faces several inherent limitations. Not all environmental genes are efficiently expressed in surrogate hosts, often leading to an underestimation of the true diversity of resistance determinants [140,142]. Most studies rely on *E. coli* as the expression host, which may fail to detect genes that require specific transcriptional or post-translational machinery present only in other bacterial taxa [143]. Additionally, overexpression artefacts, gene toxicity, and vector bias can produce false positives or mask genuine resistance functions. The method is also labour-intensive and requires subsequent validation of candidate genes to confirm their actual role in resistance [142,144].

Nevertheless, when carefully designed and combined with complementary sequencing approaches, functional metagenomics remains indispensable for discovering novel fosfomycin resistance mechanisms.

### 6.4. Quantitative and High-Throughput Detection Methods

In addition to PCR and omics-based methods, quantitative approaches such as high-throughput quantitative PCR (HT-qPCR) and digital droplet PCR (ddPCR) have significantly improved the ability to monitor ARG abundance and distribution across environmental gradients. These techniques are particularly useful for assessing the effects of agricultural practices (e.g., manure application, irrigation with reclaimed water) on the dissemination of resistance genes, including those conferring fosfomycin resistance [129,144].

HT-qPCR offers broad gene coverage and allows for the simultaneous quantification of hundreds of ARGs across multiple samples. However, this approach can be affected by primer bias and typically lacks sensitivity for genes present at low abundance. In contrast, ddPCR provides superior sensitivity, precision, and tolerance to PCR inhibitors, making it especially effective for quantifying rare resistance genes in complex environmental matrices. Nevertheless, ddPCR is characterised by lower throughput and is limited to a smaller number of targets per run. Both methods would benefit from standardised protocols and reference materials to improve data comparability and reproducibility across studies and laboratories [129,130].

### 6.5. Single-Cell Genomics and Flow Cytometry-Based Approaches

Recent advances in single-cell genomics have made it possible to link specific resistance genes to individual microbial taxa—a key limitation of bulk metagenomic approaches. Fluorescence-activated cell sorting (FACS) enables the isolation of specific bacterial subpopulations based on their physical or fluorescent properties, followed by genomic sequencing of single cells. This technique allows for the identification of fosfomycin resistance determinants within distinct microbial lineages, including unculturable species [145]. The combination of FACS and single-cell sequencing represents a promising avenue for elucidating the ecological context and potential transfer routes of ARGs in agricultural soils [146].

### 6.6. Emerging CRISPR-Based Detection

Recent innovations include CRISPR-based diagnostic platforms, such as CRISPR-Cas12a and Cas13a systems, which offer rapid, sensitive, and portable detection of resistance genes. Although their application to fosfomycin resistance remains limited, these methods hold significant promise for future field-based monitoring due to their specificity and potential for multiplexing [147,148].

The methodological landscape of fosfomycin resistance research has evolved from targeted, gene-specific assays to holistic multi-omics and single-cell approaches. Each technique offers distinct advantages and limitations, and their combined use provides the most comprehensive insight into the environmental dynamics of fosfomycin resistance. Future progress will likely depend on the integration of metagenomics, single-cell sequencing, and CRISPR-based diagnostics to enable rapid, high-resolution surveillance systems capable of tracking the spread of resistance genes across agricultural ecosystems.

## 7. Attempts to Prevent the Spread of Fosfomycin Resistance

Preventing the dissemination of fosfomycin resistance requires coordinated, multisectoral interventions embedded within the One Health framework. Although fosfomycin is considered a critically important antimicrobial in both human medicine and veterinary practice, its stewardship remains uneven across regions, and surveillance systems rarely capture its occurrence in agricultural settings. Consequently, global, regional, and national policies play essential—albeit variably effective—roles in mitigating the risk of resistance emergence and spread.

### 7.1. Global Frameworks

At the global level, the World Health Organization (WHO) provides the overarching strategic structure for AMR control through the Global Action Plan on Antimicrobial Resistance [149], the Global Antimicrobial Resistance and Use Surveillance System (GLASS) [150], and the WHO Antimicrobial Resistance Diagnostic Initiative [151]. These instruments strengthen laboratory capacity, harmonise diagnostic standards, promote responsible antimicrobial use, and integrate human, animal, and environmental data streams. Although they do not mention fosfomycin explicitly, they offer the conceptual and operational basis necessary to detect and manage resistance to this agent across sectors.

Additionally, the WHO Laboratory Assessment Tool provides a structured framework for evaluating laboratory capacity, performance, and quality, while the Stepwise Laboratory Improvement Process Towards Accreditation (SLIPTA) supports laboratories in achieving ISO 15189 standards. These tools are complementary: the WHO tool assesses operational readiness, and SLIPTA evaluates quality management and compliance with best practices. Together, they support the systematic strengthening of laboratory networks, particularly in low- and middle-income countries [152,153,154].

Complementary guidance from the WOAH addresses antimicrobial stewardship in animal husbandry. The WOAH Terrestrial Animal Health Code outlines principles for responsible antimicrobial use [155,156], while the WOAH List of Antimicrobial Agents of Veterinary Importance directly classifies fosfomycin under phosphonic acid derivatives and recommends that its use in veterinary medicine be kept at an absolute minimum due to its critical importance for human medicine. This makes it the only major global document that explicitly names fosfomycin and offers substance-specific guidance.

Importantly, the FAO/WOAH/WHO Tripartite (now Quadripartite, with the inclusion of UNEP) collaboration explicitly emphasises the need to preserve antimicrobials categorised as critical for human health by restricting their use outside human medicine whenever possible. Since fosfomycin is primarily a human therapeutic agent, used for uncomplicated urinary tract infections and as part of combination regimens for severe multidrug-resistant infections, its protection aligns directly with these global recommendations. Although most international surveillance platforms (e.g., GLASS, EFSA AMR monitoring) do not currently include fosfomycin or fosfomycin resistance genes, the Tripartite guidance positions fosfomycin as an antimicrobial for which non-essential veterinary and agricultural use should remain extremely limited. This discrepancy between its clinical importance and the absence of systematic environmental monitoring highlights a critical gap in One Health AMR surveillance [157].

### 7.2. Regional (European Union) Initiatives

Within the European Union, antimicrobial stewardship and AMR prevention are further operationalised through dedicated policy instruments, including the European One Health Action Plan [158], European Food Safety Agency (EFSA)/European Centre for Disease Prevention and Control (ECDC) [159] surveillance reports, European Medicines Agency (EMA) antimicrobial categorisation guidance, and Regulation (EU) 2019/6 on veterinary medicinal products [160]. These frameworks aim to reduce antimicrobial use in livestock, strengthen diagnostics, harmonise monitoring, and restrict the veterinary use of antimicrobials classified as critically important.

Although EU surveillance systems (e.g., EFSA AMR monitoring) do not routinely include fosfomycin susceptibility testing, they contribute indirectly to risk assessment by characterising the spread and mobility of resistance determinants relevant to fosfomycin.

### 7.3. National Action Plans and Stewardship Guidelines

Many countries have adopted National AMR Action Plans that translate global and regional guidance into locally relevant implementation strategies. These plans typically emphasise prudent antimicrobial use, control of critically important antimicrobials, improved diagnostics, enhanced surveillance, and strengthened veterinary sector oversight. However, most national action plans, irrespective of the region, do not explicitly mention fosfomycin. Instead, its regulation is embedded within broader restrictions on antimicrobials considered critical for human medicine, alongside general stewardship measures (Table 3).

In addition to policy frameworks, the regulatory landscape relevant to fosfomycin also includes several complementary domains that shape its use and monitoring across sectors. National regulatory authorities—such as the FDA (United States), PMDA (Japan), MHRA (United Kingdom), ANSM (France), and BfArM (Germany)—define the approved formulations, dosing regimens, and clinical indications for fosfomycin within their respective jurisdictions. These national approvals directly influence therapeutic use patterns and, consequently, modulate selection pressures contributing to resistance.

In the veterinary sector, Good Veterinary Practice principles developed by the WOAH, FAO, and EMA complement formal regulatory frameworks by promoting diagnostics-based therapy, restricting the use of critically important antimicrobials, and encouraging prudent antimicrobial use overall [12]. While these documents do not mention fosfomycin explicitly, their emphasis on limiting the use of human-critical antimicrobials provides an indirect layer of control over its use in animals.

Finally, environmental AMR frameworks—including the environmental modules of GLASS and EFSA’s emerging assessments of AMR in soil and water—highlight growing efforts to integrate environmental reservoirs into AMR surveillance. Although these systems do not currently monitor fosfomycin or *fos* genes specifically, they support broader risk assessments by identifying pathways through which environmental resistomes may contribute to the dissemination of resistance determinants relevant to fosfomycin.

## 8. Conclusions

Despite increasing attention to environmental AMR, no formalised or harmonised international or regional systems currently exist for monitoring fosfomycin residues or fosfomycin resistance genes (e.g., *fos*, *fosA*, *fosB*, *fosX*, *fosC2*) in agricultural soils. Existing surveillance programmes primarily focus on clinical isolates, zoonotic bacteria, or antimicrobial sales data. Where environmental monitoring exists, it remains limited in scope, non-standardised, and rarely includes fosfomycin-specific resistance determinants. This gap is particularly concerning given the demonstrated environmental reservoir function of agricultural soils, their role in facilitating horizontal gene transfer, and the documented presence of novel environmental fosfomycin resistance genes identified through metagenomics. The absence of systematic surveillance complicates risk assessment and limits the ability to detect early warning signals of fosfomycin resistance emergence at the human–animal–environment interface.

Large-scale studies focusing on fosfomycin resistance genes are urgently needed. These studies should be aimed at addressing knowledge gaps on key issues, such as the effects of pesticide exposure, nitrogen fertilisation, soil physicochemical properties, and global warming on the frequency of distribution of fosfomycin resistance genes. Several important knowledge gaps emerged during this review. The current literature provides limited information on the prevalence and mobility of fosfomycin resistance genes in soil and manure, and no standardised monitoring framework exists for the environmental monitoring of fosfomycin residues. Furthermore, the role of environmental co-selectors—such as pesticides, microplastics, heavy metals and global warming—remains insufficiently characterised, and the interaction between these stressors and the cellular processes shaping fosfomycin resistance (e.g., DNA repair, central metabolism) is poorly understood.

Future research should prioritise integrated One Health surveillance systems, functional validation of novel *fos* genes identified through meta-omics, and cross-sectoral studies within agricultural systems to better define risk pathways and identify effective intervention points.

This review contributes new insight by highlighting the under-recognised role of soil chemical stressors and environmental co-selection in shaping fosfomycin resistance within a One Health framework.

## Figures and Tables

**Table 1:** Fosfomycin resistance genes and mechanisms.

Gene/Mutation	Localisation	Encoded Protein/Function	Mechanism	Host (Example)	Ref.
Enzymatic Inactivation					
*fomA*, *fomB*	chromosome, within fosfomycin biosynthetic gene cluster	FomA, FomB (kinases)	Phosphorylates fosfomycin (FomA) and fosfomycin-phosphate (FomB)	*S. wedmorensis*	[26]
*fosA (fosA1–fosA13)*	plasmid/chromosome	FosA (glutathione S-transferase)	Mn^2+^- and K^+^-dependent; adds glutathione to open fosfomycin epoxide ring	Gram-negative bacteria (e.g., *Klebsiella*, *Enterobacter*, *Serratia*, *Pseudomonas*)	[43,44,45,46,47]
*fosB*, *fosSA*	plasmid/chromosome	FosB (bacillithiol or L-cysteine transferase)	Mn^2+^/Mg^2+^-dependent; adds thiol (bacillithiol or L-Cys) to open fosfomycinepoxide ring	Gram-positive bacteria (e.g., *Staphylococcus*)	[48,49]
*fosC*, *fosC2*, *fosC3*	plasmid/chromosome	FosC (phosphotransferase/kinase)	Uses ATP to phosphorylate fosfomycin	*E. coli*, *S. aureus*	[39,50]
*fosX*	chromosome	FosX (epoxide hydrolase)	Mn^2+^-dependent; adds water to open fosfomycin epoxide ring	*L. monocytogenes*, *C. botulinum*, *E. faecium.*	[13,50,51]
*fosL1*, *fosL2*	plasmid	FosL1, FosL2 (glutathione S-transferases)	FosA-like enzymatic activity	Enterobacteriaceae	[52]
*fosD*	chromosome	FosD (bacillithiol transferase)	Adds bacillithiol to open fosfomycin epoxide ring	*S. aureus*	[51]
*fosY*	chromosome	FosY (putative bacillithiol transferase)	FosB-like enzymatic activity	*S. aureus* (CC1 MRSA)	[53]
*fosSC*	chromosome	FosSC (putative bacillithiol transferase)	FosB-like enzymatic activity	*S. capitis*	[54]
*fosK*	chromosome	FosK (glutathione S-transferase)	FosA-like enzymatic activity	*Acinetobacter soli*	[41]
*fosM*	chromosome	FosM (bacillithiol transferase)	FosB-like enzymatic activity	*Bacillus*	[42]
*murA*	chromosome	MurA (UDP-N-acetylglucosamine enolpyruvyl transferase)	Makes MurA active site inaccessible through a cysteine-to-aspartate mutation	*E. coli*, *S. aureus*	[48,55,56,57]
**Transportation**					
*glpT*	chromosome	GlpT (glycerol-3-phosphate transporter)	Mutation in the gene leads to inactivation of the transporter		[20,58,59]
*uhpT*	chromosome	UhpT (Hexose-6-phosphate:phosphate antiporter	Mutation in the gene leads to inactivation of the transporter		
*uhpA*, *uhpB*, *uhpC*	chromosome	*uhpT* regulatory genes	Mutation in the gene reduces the expression of *uhpT*	*E. coli*, *S. aureus*	[20,58,59]
*ptsI*	chromosome	responsible for cAMP synthesis, uhpT and glpT regulatory gene	Mutation in the gene reduces intracellular levels of cAMP and subsequently expression of *glpT* and *uhpT*	*E. coli*	[60]
*cyaA*	chromosome	responsible for cAMP synthesis, *uhpT* and *glpT* regulatory gene	Mutation in the gene reduces intracellular levels of cAMP and subsequently expression of *glpT* and *uhpT*	*E. coli*	[60]
*cpxA*, *cpxR*	chromosome	*uhpT* and *glpT* regulatory gene	Deletions in the *cpxA* gene result in constitutive expression of its regulator *cpxR*. CpxR directly represses *glpT* and *uhpT* expression	*E. coli*	[61]
*tet38*	chromosome	Tet38 efflux transporter	Facilitates fosfomycin removal from cytoplasm	*S. aureus*	[62]
**Related with central metabolism**					
*mutL*, *mutS*	chromosome	DNA mismatch repair system	Mutation in these genes impair DNA repair mechanisms leading to the accumulation of mutations that confer fosfomycin resistance	*E. coli*	[59]
*purB*	chromosome	Adenylosuccinate lyase/de novo purine nucleotide biosynthesis	Requires further investigation, putative role in cAMP synthesis	*E. coli*	[59]
*hflD*	chromosome	lysogenisation regulator (upstream of purB)	Requires further investigation	*E. coli*	[59]

**Table 2:** Fosfomycin resistance genes detected in agriculture-related samples.

Host Microorganism	Source	Resistance Gene	Ref.
*K. pneumoniae and K. variicola*	food	*fosA*	[13]
*E. coli*	chicken, farms in China	*fosA3*	[30]
*E. coli* ABW A19 (ST1266), ESBL DR28 (ST167); SBF22 (ST354)	wastewater, food and surface water	*fosA3*	[68]
*E. coli*	broiler chickens and poultry farm environmental samples	*fosA3*	[69]
*E. coli*	food animals (pigs, chickens, pigeons) and their environments (China)	*fosA3*	[70]
*E. coli*	isolates obtained from pigs, chickens, dairy cows, and staff (China)	*fosA3*	[71]
*Raoultella ornithinolytica*, *E. coli*, *K. pneumoniae*, *Citrobacter freundii*	vegetables: lettuce, cucumber, tomato, bean sprouts,	*fosA3*	[72]
*E. coli*	retail food: in beef	*fosA3*	[73]
*E. coli*	faecal samples collected at Brazilian broiler farms	*fosA3*	[74]
*E. coli*	faecal droppings of wild birds in the urban parks in Faisalabad, Pakistan	*fosA4*	[75]
*E. coli*	retail food: chicken meet	*fosA4*	[73]
*K. pneumoniae*	unpasteurised raw milk samples	*fosA5*	[29]
*K. pneumoniae*	raw milk (Egypt)	*fosA5*	
*S. enterica*	food animals and retail meat products in China	*fosA7*	[76]
*S. enterica serovar Heidelberg*	broiler chicken	*fosA7*	[45]
*E. coli*	bovine from USA	*fosA7*,*5* (variant of *fosA7 gene*)	[77]
*E. coli*	isolates from pig, chicken and pigeon in China	*fosA7*,*5* (variant of *fosA7 gene*)	[70]
*K. pneumoniae ESBL DR09 (ST307)*	food	*fosA8*	[68]
*E. coli isolate PK9*	recovered from a chicken meat (China)	*fosA10*	[28]
	waterfowl (China)	*fosA10*	[31]
*M. morganii isolate DW0548*	poultry on a farm in Wenzhou, China	*fosA13*	[31]
*Aeromonas caviae DW0021*	soil sample from an animal farm in Wenzhou, China	*fos*C3, *fosSC*, *fosG*, *fosL1*, *FosL2*, *Orf1*	[39]
*S. aureus*.	duck farms (China)	*fosB*	[78]
*E. faecalis*	from pigs	*fosB*	
*S. aureus*	chicken and pork isolates (United States)	*fosB*	[79]
*Bacillus cereus*	house crickets	*fosB1–B3*	[80]
*S. arlettae strain SA-01*	chicken farm in China	*fosD*	[81]
*S. haemolyticus*	swine slaughterhouse	*fosS*	[82]

**Table 3:** Global and regional strategies aimed at reducing AMR spread through agriculture.

Framework/Policy/Initiative	Main Recommendations and Actions	Relevance to Fosfomycin Use	Key Limitations/Challenges	Ref.
Global				
WHO Global Action Plan on Antimicrobial Resistance	Strengthen AMR surveillance, optimise antimicrobial use, support innovation, and improve awareness under the One Health framework.	Provides a strategic One Health framework for reducing antimicrobial misuse and preserving critically important antimicrobials, which indirectly includes fosfomycin despite not being mentioned by name.	Does not address fosfomycin explicitly; recommendations remain broad and require national-level translation to affect specific antimicrobials.	[149]
WHO GLASS Global Antimicrobial Resistance and Use Surveillance System	Establishes standardised global surveillance of AMR and antimicrobial use; supports laboratory capacity, harmonised methodologies, and data reporting; integrates human, animal and (pilot) environmental surveillance modules.	Indirect relevance: GLASS establishes harmonised surveillance of priority pathogens and critically important antimicrobials; but fosfomycin is not part of its standard reporting panels. Data generated by GLASS can support broader assessments of AMR trends in bacterial reservoirs that may also harbour *fos* genes.	Fosfomycin is not monitored; environmental sampling remains limited; uneven diagnostic capacity across countries restricts comparability.	[150,161]
WHO Antimicrobial Resistance Diagnostic Initiative	Expand diagnostic capacity, standardise laboratory systems, and ensure access to reliable AMR testing.	Supports improved laboratory capacity and diagnostic accuracy for AMR detection, which enhances the ability to identify fosfomycin-resistant isolates even though the drug is not targeted specifically.	Does not include drug-specific recommendations; focuses on system capacity rather than antimicrobial classes; implementation is constrained in low-resource settings.	[151]
WOAH Terrestrial Animal Health Code	Promote prudent and responsible antimicrobial use in animals; ban antibiotics as growth promoters; reinforce veterinary oversight.	Provides global principles for prudent antimicrobial use in animals, which apply equally to fosfomycin as a critically important antimicrobial for human medicine.	Does not mention fosfomycin by name; focuses on general stewardship principles rather than substance-specific restrictions.	[155]
WOAH List of Antimicrobial Agents of Veterinary Importance	Provide classification of antimicrobials for veterinary use, including a recommendation that fosfomycin use should be kept at absolute minimum.	Explicitly lists fosfomycin (cyclic esters) and recommends minimal use in veterinary medicine due to high resistance risk.	Implementation varies across member states; document focuses on classification rather than enforcement; environmental compartments not addressed.	[7]
**Regional (EU)**				
European One Health Action Plan against Antimicrobial Resistance	Reduce antimicrobial use in livestock by 50%; strengthen AMR surveillance; support R&D and stewardship programmes.	Includes monitoring of fosfomycin use in livestock, aquaculture, and zoonotic pathogens.	Variable national implementation; data gaps in environmental compartments.	[158]
EU Council Recommendation on stepping up EU actions to combat AMR	Strengthen One Health national action plans, reinforce surveillance/monitoring of AMR and antimicrobial consumption, set targets for AMU, improve awareness and training.	Supports the reduction in antimicrobial use in the animal and human farming sectors; indirectly supports better management of important antibiotics, including fosfomycin.	Diverse implementation across Member States; surveillance gaps especially in environmental sector.	[162]
EFSA/ECDC European Union Summary Report on Antimicrobial Resistance in zoonotic and indicator bacteria	Provides comprehensive surveillance data on AMR in zoonotic (e.g., *Salmonella*, *Campylobacter*) and indicator bacteria (e.g., *E. coli*) from animals, food, and the environment; identifies emerging resistance trends; supports risk assessment and evidence-based policy decisions across the EU.	May be indirectly relevant for fosfomycin resistance, as it monitors key bacterial reservoirs (e.g., commensal *E. coli*, ESBL/AmpC producers) that commonly harbour plasmid-mediated *fos* genes, supporting broader risk assessment of potential transmission along the food chain.	Fosfomycin is not included in the harmonised EU AMR monitoring panel; environmental compartments (soil and water) remain largely unmonitored, and methodological variability across Member States limits comparability.	[163]
EMA/EFSA Categorisation of Antimicrobials (2017)	Provides scientific advice on antimicrobial classes and categorisation by risk to human health; includes Appendix I listing CIAs authorised in human medicine only.	Includes Appendix I specifying that cyclic esters such as fosfomycin are CIAs authorised for human medicine only, and their use in veterinary practice should be kept at an absolute minimum due to the high risk of resistance dissemination.	Based on data available up to 2017; categorisation not updated recently.	[159]
EU Regulation (EU) 2019/6 on Veterinary Medicinal Products	Regulation lays down general rules to restrict the use of antimicrobials of critical importance for human medicine in animals, including the possibility of reserving such substances for human use only.	Although fosfomycin is not specified by name, the regulation’s provisions on reserving critical antimicrobials for humans provide a legal basis for restricting veterinary use of fosfomycin.	No specific mention of fosfomycin; implementation relies on national legislation and monitoring of veterinary antimicrobials sales; environmental compartments not explicitly addressed.	[160]
Veterinary Guidelines/International and Regional				
Good Veterinary Practice (FAO, EMA)	Promotes targeted therapy, diagnostics, and reduction in CIAs	Indirectly restricts veterinary fosfomycin use.	Does not name fosfomycin explicitly; adoption varies.	[12,164,165]

## Data Availability

No new data were created or analyzed in this study. Data sharing is not applicable to this article.

## Abbreviations

The following abbreviations are used in this manuscript:

AMR	antimicrobial resistance
ANSM	The French National Agency for Medicines and Health Products Safety
BfArM	Federal Institute for Drugs and Medical Devices
ddPCR	digital droplet PCR
EC	European Comission
ECDC	European Centre for Disease Prevention and Control
EFSA	European Food Safety Agency
EMA	European Medicines Agency
EU	European Union
FACS	Fluorescence-activated cell sorting
FAO	Food and Agriculture Organization
FDA	Food and Drug Administration
GLASS	WHO Global Antimicrobial Resistance and Use Surveillance System
HPCIA	Highest Priority Critically Important Antimicrobials
MHRA	Medicines and Healthcare products Regulatory Agency
OPs	organophosphonates
PMDA	Pharmaceuticals and Medical Devices Agency
SLIPTA	Stepwise Laboratory Improvement Process Towards Accreditation
UNEP	The United Nations Environment Programme
WHO	World Health Organization
WOAH	World Organisation for Animal Health
